# *In vivo* biocompatibility testing of nanoparticle-functionalized alginate–chitosan scaffolds for tissue engineering applications

**DOI:** 10.3389/fbioe.2023.1295626

**Published:** 2023-11-23

**Authors:** Nancy G. Viveros-Moreno, Mario Garcia-Lorenzana, Eduardo Peña-Mercado, Josune García-Sanmartín, Judit Narro-Íñiguez, Marcela Salazar-García, Sara Huerta-Yepez, Concepción Sanchez-Gomez, Alfredo Martínez, Nohra E. Beltran-Vargas

**Affiliations:** ^1^ Doctorado en Ciencias Biológicas y de la Salud, Universidad Autonoma Metropolitana, Ciudad de Mexico, Mexico; ^2^ Department of Reproduction Biology, Division of Biological and Health Sciences, Universidad Autonoma Metropolitana, Iztapalapa, Mexico; ^3^ Department of Processes and Technology, Division of Natural Sciences and Engineering, Universidad Autonoma Metropolitana, Cuajimalpa, Mexico; ^4^ Angiogenesis Group, Center for Biomedical Research of La Rioja (CIBIR), Logroño, Spain; ^5^ Research Laboratory of Developmental Biology and Experimental Teratogenesis, Children’s Hospital of Mexico Federico Gomez, Mexico City, Mexico; ^6^ Research Laboratory of Hematooncology, Children’s Hospital of Mexico Federico Gomez, Mexico City, Mexico

**Keywords:** alginate, chitosan, biocompatibility, foreign body reaction, subcutaneous implantation

## Abstract

**Background:** There is a strong interest in designing new scaffolds for their potential application in tissue engineering and regenerative medicine. The incorporation of functionalization molecules can lead to the enhancement of scaffold properties, resulting in variations in scaffold compatibility. Therefore, the efficacy of the therapy could be compromised by the foreign body reaction triggered after implantation.

**Methods**: In this study, the biocompatibilities of three scaffolds made from an alginate–chitosan combination and functionalized with gold nanoparticles (AuNp) and alginate-coated gold nanoparticles (AuNp + Alg) were evaluated in a subcutaneous implantation model in Wistar rats. Scaffolds and surrounding tissue were collected at 4-, 7- and 25-day postimplantation and processed for histological analysis and quantification of the expression of genes involved in angiogenesis, macrophage profile, and proinflammatory (IL-1β and TNFα) and anti-inflammatory (IL-4 and IL-10) cytokines.

**Results**: Histological analysis showed a characteristic foreign body response that resolved 25 days postimplantation. The intensity of the reaction assessed through capsule thickness was similar among groups. Functionalizing the device with AuNp and AuNp + Alg decreased the expression of markers associated with cell death by apoptosis and polymorphonuclear leukocyte recruitment, suggesting increased compatibility with the host tissue. Similarly, the formation of many foreign body giant cells was prevented. Finally, an increased detection of alpha smooth muscle actin was observed, showing the angiogenic properties of the elaborated scaffolds.

**Conclusion**: Our results show that the proposed scaffolds have improved biocompatibility and exhibit promising potential as biomaterials for elaborating tissue engineering constructs.

## 1 Introduction

There is a great interest in developing novel scaffolds in tissue engineering (TE) (Goldenberg et al., 2021; Bertsch et al., 2023; Han et al., 2023). To maintain cell viability and functionality, biomaterials used as scaffolds must satisfy biophysical and biochemical requirements associated with mechanical strength, porosity, biodegradability, and biocompatibility (Dzobo et al., 2018). Implant devices often have compromised efficacy due to host recognition problems and subsequent responses, resulting in acute inflammation, chronic inflammation, granulation tissue, foreign body reaction (FBR), chronic encapsulation, or dissolution of the implanted biomaterial (Veiseh et al., 2015; Chung et al., 2017; Ibrahim et al., 2017; Carnicer-Lombarte et al., 2021; Wei et al., 2021).

Immune recognition of a biomaterial initiates a cascade of cellular processes leading to FBR. The response to the materials occurs in four phases: hemostatic, inflammatory, proliferative, and remodeling. Degradation or even complete phagocytosis of the biomaterial resolves the FBR. A failed transition from the inflammatory to the proliferative phase leads to a failed resolution, characterized by fibrous encapsulation rather than tissue regeneration. During this transition, immune cells, such as macrophages and neutrophils, play a crucial role by altering their phenotype and recruiting cells that will follow in the proliferative phase (Anderson et al., 2008; Major et al., 2015; Chung et al., 2017; Martin and García, 2021).

The intensity of the inflammatory response is mainly determined by the composition of the biomaterial and by the porosity, hydrophobicity, topography, and biodegradability of the scaffold, which lead to the recruitment and reactivity of cellular mediators after implantation (Abaricia et al., 2021; Martin and García, 2021; Kyriakides et al., 2022).

Biomaterials of natural origin have been documented to cause mild FBR relative to those of synthetic origin (Ibrahim et al., 2017). Porosity has been shown to impact FBR positively. Porous scaffolds (>40 µm) elicit less severe inflammatory responses (Veiseh et al., 2015), by polarizing macrophages towards the M2 phenotype. Also, porosity contributes to increased vascularization, cellular infiltration, and reduced fibrosis (Kyriakides et al., 2022; Li et al., 2022). Hydrophobicity plays an important role in the degradation of biomaterials and in the adsorption of proteins on the biomaterial. Depending on the hydrophobicity, proteins will have different affinities for the biomaterial, resulting in different inflammatory responses (Jeong et al., 2017). Scaffolds with hydrophilic ends have been documented to result in increased expression of anti-inflammatory cytokines, M2 macrophage recruitment, optimal tissue infiltration (Flaig et al., 2020), and increased material-cell interaction (Patil et al., 2022). The topography of the biomaterial may also affect the FBR, specifically regarding macrophage behavior (Witherel et al., 2019). Finally, it has been observed that biomaterials with prolonged tissue residence develop a relatively avascular collagen-rich capsule around the implant, which sequesters it from the surrounding tissue (Ibrahim et al., 2017).

Strategies aimed at interfering with cellular events driving FBR have been proposed in the design of bioactive scaffolds (Abaricia et al., 2021), including immunomodulatory biomaterials (Whitaker et al., 2021; Chen et al., 2022), functionalization of the scaffold with anti-inflammatory molecules, or with optimization and conservation of bioactive components that maximize the bioactive potential of the biomaterial (Joyce et al., 2021). Thus, the scaffold design should support cellular activity without hindering the post implantation signaling cascade.

Natural biomaterials possess bioactive properties so that biological activity can be imparted to a material using natural polymers (Joyce et al., 2021). Chitosan and alginate stand out among the vast array of natural biomaterials. Chitosan (Cs)—a natural polysaccharide made from glucosamine and an N-acetyl-glucosamine moiety—is extracted from crustacean shells through deacetylation. Cs has the highest chelating capacity of all natural polymers and promotes cell adhesion, proliferation, and differentiation (Muxika et al., 2017; Joyce et al., 2021). Alginate (Alg) is a natural polysaccharide found in marine algae, which contains linked blocks of β-D-mannuronic acid (M) and α-L-guluronic acid (G) monomers (1–4). Alg exhibits poor cell adhesion but combined with peptides or other polymers, such as Cs, it enhances cell adhesion and proliferation *in vitro*. Alg is a biomaterial capable of incorporating and retaining cells and proteins (Sun and Tan, 2013; Joyce et al., 2021) and promotes angiogenesis (Sondermeijer et al., 2018).

Since the search for strategies to improve the electrical properties of biomaterials began, using metallic nanostructures, such as gold (Au), has become relevant in TE (Yadid et al., 2019). It has been reported that the incorporation of Au nanoparticles (Np) reduces apoptosis and inflammation (Shevach et al., 2014; Sridhar et al., 2015; Somasuntharam et al., 2016), which is conducive to cell proliferation (Maharjan et al., 2019), in addition to improving the physical properties of the scaffold (Yadid et al., 2019).

Our working group has designed scaffolds for applications in TE using sodium Alg and Cs, functionalized with alginate-coated gold nanoparticles (AuNp + Alg). The resulting scaffolds are highly porous (>90%) and hydrophilic, with swelling percentages of approximately 3,000% and permeability in the order of 1 × 10^−8^ m^2^ (Beltran-Vargas et al., 2022). Although a physicochemical characterization of the proposed scaffolds was carried out and cell growth tests were reported, with better results using AuNp + Alg, it is important to study how this novel scaffold affect host response, such as inflammation and immune modulation *in vivo*.

This work aimed to analyze the biocompatibility of three types of Alg/Cs scaffolds with and without AuNp functionalization by subdermal implantation in Wistar rats.

## 2 Materials and methods

### 2.1 Scaffolding

Sodium alginate (Sigma Aldrich, Mannheim, Germany, #9005-38-3) and chitosan (medium molecular weight, Sigma Aldrich, Mannheim, Germany, #448877) (0.75%–1.25% w/v) powder were mixed and dissolved in ultrapure water and acetic acid (1% w/v, Sigma Aldrich). pH was adjusted between 5 and 6. The solution was placed into 24-well plates. After freezing and freeze-drying, cross-linking was performed with 1% calcium gluconate for 30 min. Subsequently, washings were performed with ultrapure water, and the mixture was dried and freeze-dried for 8 h. Functionalization of Alg/Cs scaffolds with gold nanoparticles (AuNp) was performed as previously reported (Beltran-Vargas et al., 2022).

The scaffolds have 1.4 cm in diameter, 12 mg in weight, and 0.3 cm wide, with 93% swelling, referred to the maximum swelling of the scaffolds, after 40 min of contact with aqueous medium, more than 90% porosity, and degrades less than 20% after 7 days. The average diameter of AuNp was 74.5 and 91 nm for AuNp + Alg. The surface charge values were in average −25.5 and −37 mV for AuNp and AuNp + Alg respectively. AuNp presented a spheroidal structure whereas AuNp + Alg showed cylindrical particle characteristics (Beltran-Vargas et al., 2022).

Unfunctionalized Alg/Cs scaffolds (without Np), Alg/Cs scaffolds functionalized with gold nanoparticles (AuNp), and Alg/Cs scaffolds functionalized with alginate-coated gold nanoparticles (AuNp + Alg) were obtained.

### 2.2 *In vivo* subcutaneous model

The experiments were performed with female (250–300 g) and male (300–350 g) Wistar rats (*n* = 4 per group), which were provided by the biotherium of the Federico Gomez Children’s Hospital of Mexico. The rats were kept in a controlled environment (22°C ± 2°C) with 50%–60% relative humidity and 12–12 h light–dark cycles, with access to food and water *ad libitum* until surgery. All animal procedures follow protocols strictly conformed by Mexican Official Guidelines (NOM-062-ZOO-1999) and were approved by the research, ethics, and biosafety committees of the Children’s Hospital of Mexico Federico Gomez (HIM/2020/059).

**FIGURE 1:**
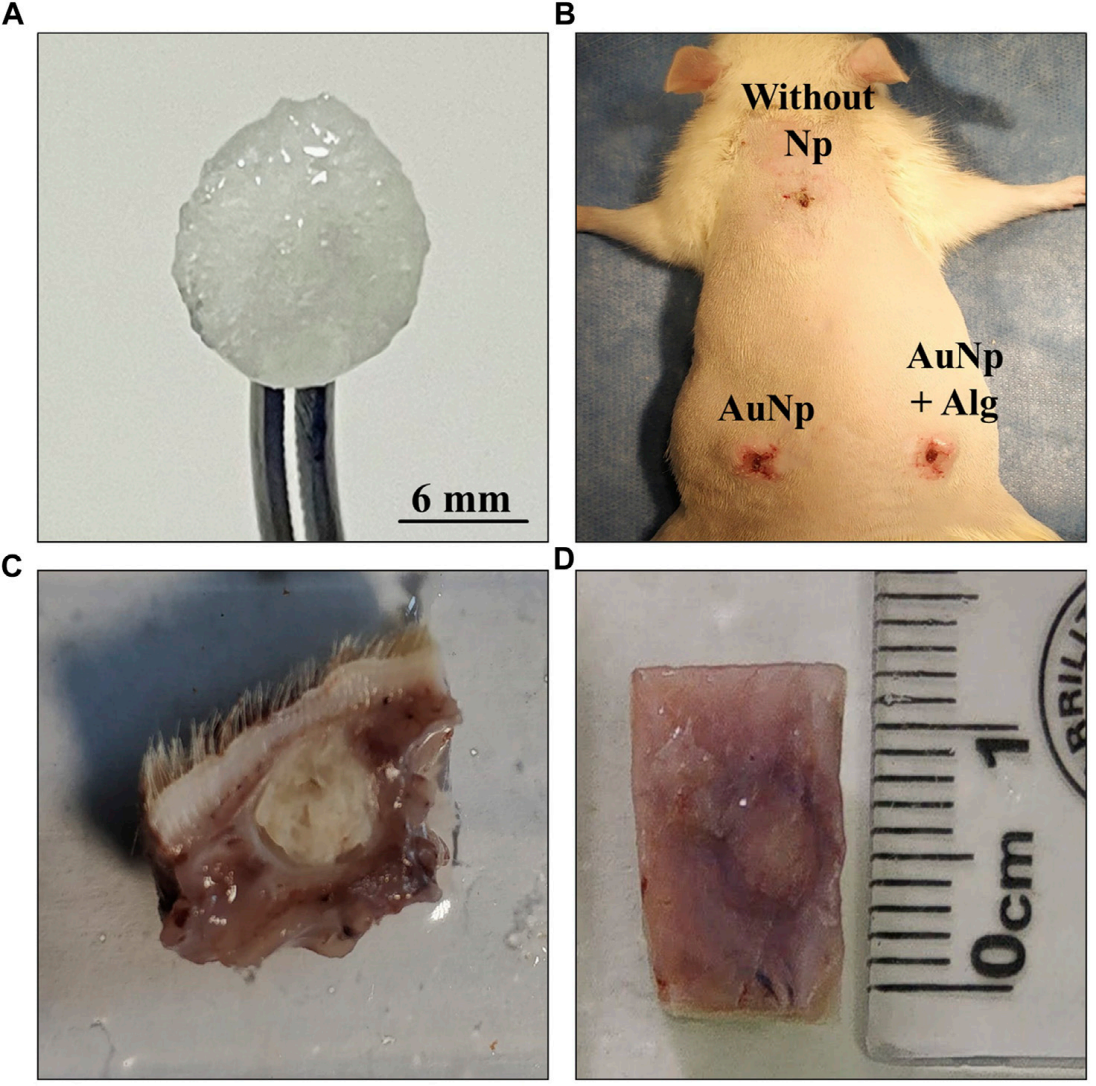
**(A)** Macroscopic appearance of a hydrated chitosan–alginate scaffold. **(B)** Subdermal implantation sites in the dorsal region, no signs of infection or rejection are appreciated. **(C, D)** General view of tissues collected after sacrifice.

Subcutaneous implantation of the scaffolds was performed through three 1-cm incisions in the dorsum of the rat under aseptic conditions (70% ethanol) and anesthesia (xylazine and ketamine (10–90 mg/kg) administered intraperitoneally. Each specimen received a scaffold without Np in the interscapular area and functionalized with AuNp and AuNp + Alg on the sides. Prior to implantation, the scaffolds were hydrated for 24 h in phosphate-buffered saline under sterile conditions, and their final dimensions were 6 mm diameter × 0.1 mm thick. A subcutaneous pocket was formed between the skin and muscle tissue, and the corresponding scaffold was placed. After implantation, the incisions were closed with surgical glue (Vetbond Tissue Adhesive 1469Sb) (Figures 1A, B).

The animals were sacrificed on days 4, 7, and 25 after implantation, and the implants were obtained with the surrounding tissue (Figures 1C, D). Four animals were used for each trial. At the end of the experiments, the animals were sacrificed according to NOM-062-ZOO-1999.

### 2.3 Histological procedure

Tissue samples were fixed in 4% neutral formalin (pH 7.4). The tissue was then processed with standard histological technique and embedded in Paraplast Plus. Finally, 3-µm thick serial transverse sections were made with a rotating microtome and premounted to apply different staining techniques. The overall architecture and infiltration of cells in the connective tissue were observed with hematoxylin-eosin (H–E) staining, and fibrotic tissue development was observed with Masson’s trichrome (MT) stain.

### 2.4 Cell infiltrate and identification of foreign body giant cells

Cell infiltration was determined with H–E staining to quantify cell migration into the scaffold for 4, 7, and 25 days. Six fields per scaffold were scanned and digitized (×20 objective) with Aperio CS2 equipment (Leica Biosystems, Deer Park, IL, United States). Quantitative analysis of the nuclei of infiltrating cells was performed with ImageJ software (National Institutes of Health [NIH]). Photomicrographs were separated into layers via Split Channels. Nuclei within the scaffold were isolated via “color thresholding.” The tool “analyze particles” was used to quantify nuclei within the scaffold boundaries. To ensure that the analysis was objective, all samples were quantified with the same thresholding conditions as reported by (Dulany et al., 2020). Subsequently, the average number of infiltrating cell nuclei was obtained for each scaffold type.

For quantification of foreign body giant cells (FBGCs), six fields per scaffold were used. Sections were photographed with ×20 objective, and the total number of cells identified per field was recorded for analysis.

### 2.5 Fibrotic capsule identification

MT stain was used to identify fibrotic capsule formation around the perimeter of the implanted scaffolds. The fibrotic capsule was determined by the presence of dense collagen bands positive for aniline blue at 4- and 7-day postimplantation. Fibrotic tissue thickness was recorded in 500 µm fields using the Aperio software “pencil” tool to quantify capsule thickness. On average, 25 measurements were obtained per specimen, which were averaged to determine the thickness of the fibrotic capsule.

### 2.6 Evaluation of collagen deposits internal to the scaffold

Photomicrographs with MT stain of the general field obtained at 25 days were taken with Aperio Software and analyzed using Fiji-ImageJ. With the “Color Deconvolution” tool, the “vectors = Brilliant_Blue” was obtained. The scaffold was delimited with this layer, and the remnants of the capsule and surrounding tissue were excluded. Finally, the “analyze particles” tool was used to quantify the area occupied by the collagen deposits in this region.

### 2.7 RNA extraction, reverse transcription, and real-time polymerase chain reaction (PCR)

Total RNA was isolated from paraffin tissues section (15 µm-thick) and purified with the High Pure FFPE RNA Isolation kit (Roche Diagnostics, Indianapolis, IN), according to manufacturer’s instructions. Resulting RNA (1.0 µg) was reverse transcribed using the NZY First-Strand cDNA Synthesis kit (Nzytech, Lisboa, Portugal), and the synthesized cDNA was amplified using NZY Supreme qPCR Green Master Mix (Nzytech). Transcripts were amplified by real-time PCR (QuantStudio 5, Applied Biosystems, Waltham, MA, United States) as described (García-Sanmartín et al., 2022). A specific cDNA calibration curve was included. GAPDH was used as a housekeeping gene (Table 1).

**TABLE 1:** Sequence of the primers used for quantitative Reverse Transcription—Polymerase Chain Reaction (qRT-PCR) and their annealing temperature.

Gene of interest	Sense primer	Antisense primer	Annealing temp.
*TNFalpha*	CCA​CCA​CGC​TCT​TCT​GTC​TA	CAC​TTG​GTG​GTT​TGC​TAC​GA	60°C
*CD11c*	AGA​AGG​GGA​CAG​GTT​GGA​CT	GCC​TGG​ACT​GTG​CTT​GGT​AA	60°C
*Tlr4*	TCT​CAC​AAC​TTC​AGT​GGC​TGG	AGT​ACC​AAG​GTT​GAG​AGC​TGG	60°C
*iNOS*	AGG​CCA​CCT​CGG​ATA​TCT​CT	GCT​TGT​CTC​TGG​GTC​CTC​TG	60°C
*CD86*	CTT​ACG​GAA​GCA​CCC​ACG​AT	TGT​AAA​TGG​GCA​CGG​CAG​AT	60°C
*IL-4*	TCC​ACG​GAT​GTA​ACG​ACA​GC	TGG​TGT​TCC​TTG​TTG​CCG​TA	60°C
*IL10*	AGG​CGC​TGT​CAT​CGA​TTT​CT	CTC​TTC​ACC​TGC​TCC​ACT​GC	60°C
*VEGFa*	CCA​GGC​TGC​ACC​CAC​GAC​AG	CGC​ACA​CCG​CAT​TAG​GGG​CA	60°C
*L1b*	AGG​CTG​ACA​GAC​CCC​AAA​AG	CTC​CAC​GGG​CAA​GAC​ATA​GG	60°C
*Arg1*	CTC​CAA​GCC​AAA​GCC​CAT​AG	GCT​GCG​GGA​CCT​TTC​TCT​AC	60°C
*Mrc1/CD206*	CAA​GGA​AGG​TTG​GCA​TTT​GT	GGA​ACG​TGT​GCT​CTG​AGT​TG	60°C
*Pecam1*	AGC​ACA​CAG​AGA​GCT​TCG​TC	TTT​GTC​CAC​GGT​CAC​CTC​AG	60°C
*Gapdh*	ATG​GTG​AAG​GTC​GGT​GTG​AAC	TCT​CAG​CCT​TGA​CTG​TGC​C	60°C

Healthy skin was used as a control of the experiment.

### 2.8 Immunohistochemistry

Tissue sections (3 µm-thick) were dewaxed in xylene, and endogenous peroxidase was blocked with 3% H_2_O_2_ in methanol for 15 min. Samples were rehydrated and subjected to antigen retrieval (10 mM Sodium Citrate, 0.5% Tween 20, pH 6.0, 20 min at 95°C). Nonspecific binding was blocked by exposure to the protein block buffer (Novocastra Leica Biosystems, Newcastle, UK) for 30 min. Then tissue sections were incubated with rabbit polyclonal antibody against Iba1 (019-19741, FUJIFILM Wako Chemicals United States corporation), at 1:500 dilution or with mouse monoclonal antibody against α-SMA (a2547, Sigma-Aldrich), at 1:5,000 dilution at 4°C, overnight.

The following day, sections were incubated with post-primary solution and Novolink polymer (Novocastra Leica Biosystems, Wetzlar, Germany), followed by exposure to 3,3′-diaminobenzidine (Dako, Carpinteria, CA, United States). Slides were lightly counterstained with hematoxylin and analyzed with an Eclipse 50i microscope (Nikon, Tokyo, Japan) equipped with a DXM 1200c digital camera (Nikon).

Quantification of immunohistochemical signals. At least six images from each stained section of each sample were analyzed. Immunoreactivity was evaluated using the ImageJ free software (NIH, Bethesda, MD), following published guidelines (Crowe and Yue, 2019). The procedure included the selection of the region of interest, color deconvolution, threshold setting, and measurement of fraction area (percentage of pixels highlighted in red from the selected area).

### 2.9 Statistical analysis

Normality of the dataset distribution was assessed using the one-sample Kolmogorov–Smirnov test. Infiltrated cell area, cell density, and capsule thickness analysis were performed with one-way analysis of variance test followed by a Tukey *post hoc* T3 (Six fields per scaffold were scanned and digitized for those analysis). Since the number of animals were small and the distribution was not normal in the other variables analysed, those datasets were compared with the Kruskal–Wallis test. A *p*-value<0.05 was considered statistically significant. Data are presented as the mean ± standard error of the mean (SEM). Analyses were performed using Prism, version 9 (GraphPad Software, San Diego, CA, United States).

## 3 Results

### 3.1 Postimplantation macroscopic observations

All scaffolds remained at the original implantation site with no apparent signs of infection, rejection, tissue necrosis, or abscess formation around the scaffolds. Detailed identification shows a lack of calcifications in the connective tissue (Figures 1B–D).

### 3.2 Biocompatibility and cellular infiltration in alginate–chitosan scaffolds

**FIGURE 2:**
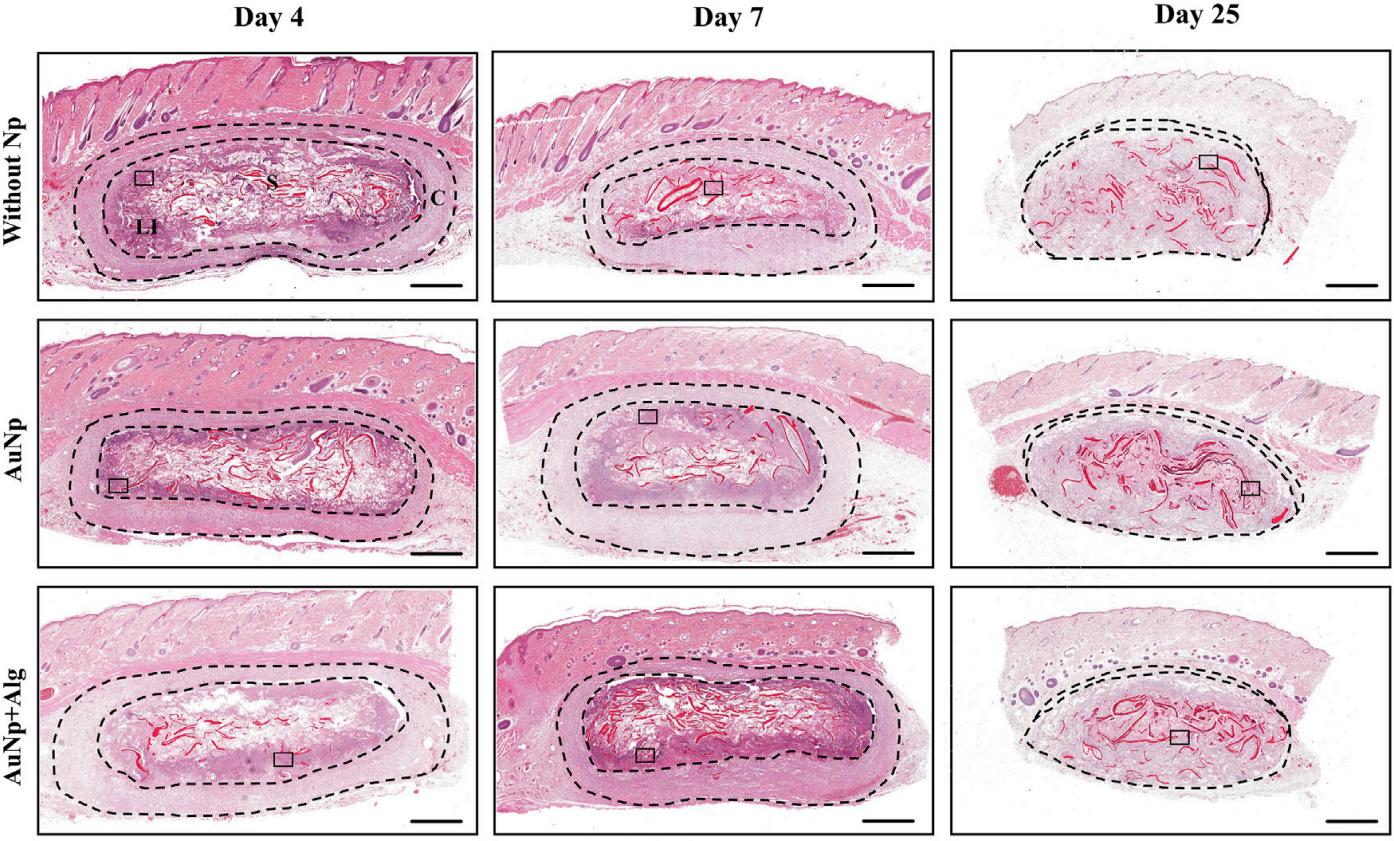
Representative photomicrographs of subcutaneously implanted chitosan-alginate scaffolds showing FBR and the development and evolution of the capsule over time, (H–E; bar = 1 mm). Identifiers: C: capsule, LI: leukocyte infiltrate, S: scaffold. The thickness of the capsule is shown between the dashed lines. After 25 days, the capsule decreases. Framed regions were enlarged in Figure 3 to show the details of the cellular infiltrates.

Scaffold biocompatibility and cellular infiltration were examined using H–E staining 4, 7, and 25 days after implantation. The overall fields of the longitudinal section of representative scaffolds per group are shown in Figure 2. The host tissue reaction to implantation is consistent with FBR, characterized by the formation of a capsule surrounding the material and the recruitment of immune cells (Figure 2). Within the global view, it was observed that the scaffolds maintain their overall shape throughout the study. The pores of the scaffold are occupied by leukocyte infiltrate, which, over time, gets homogeneously distributed in the center of the scaffold. Resolution of the event at 25 days includes dissolution of the capsule without completely degrading the scaffold. A granulation tissue remains in place in the capsule.

**FIGURE 3:**
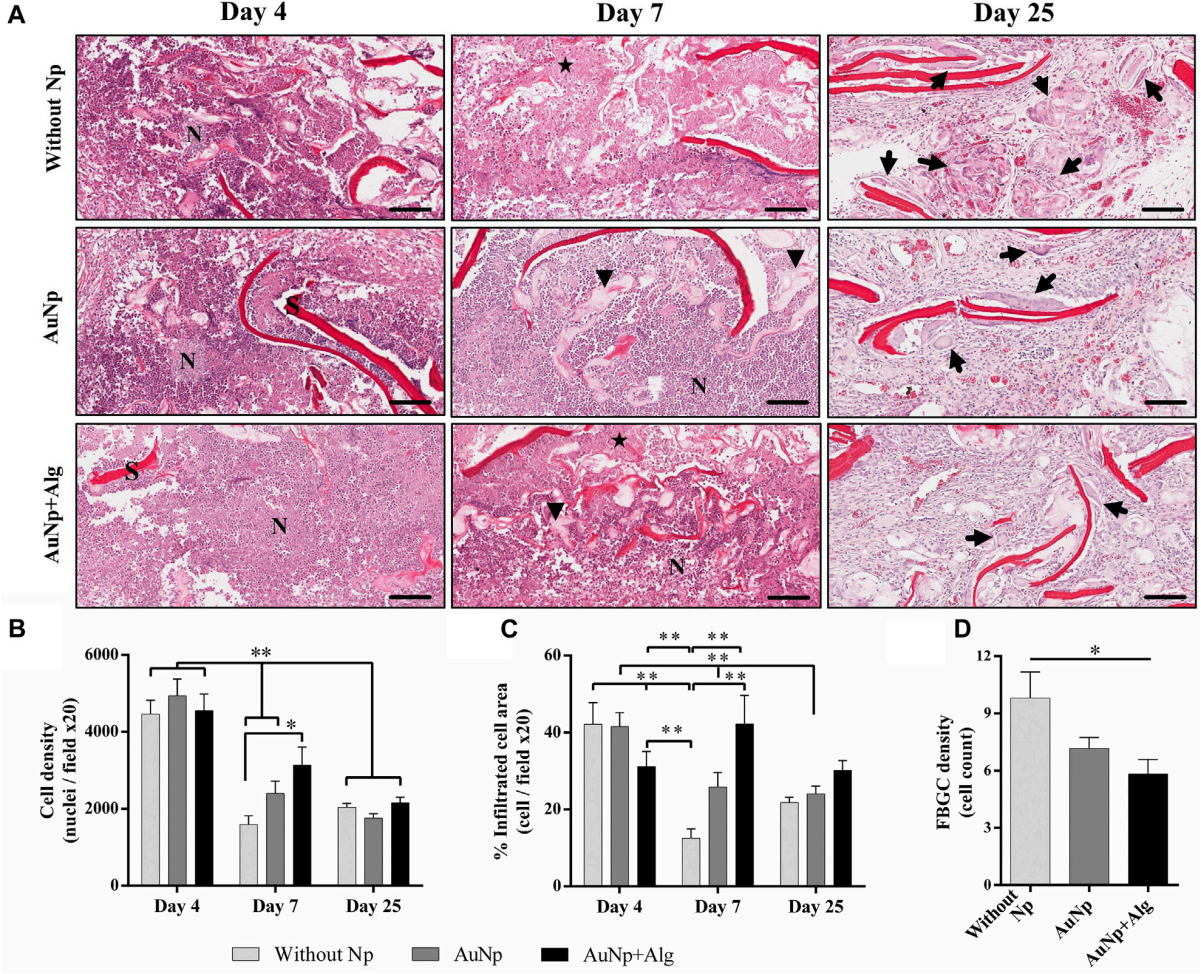
**(A)** Characterization of the response to a foreign body, (H-E; bar = 100 µm). Identifiers: Arrow: foreign body giant cells; arrowhead: myxoid areas; N: neutrophils; star: necrosis; S: scaffold. Graphical representation of **(B)** cell density, **(C)** percentage of area occupied by the cellular infiltrate, and **(D)** density of foreign body giant cells. *p < 0.01, **p < 0.001. Data are presented as mean ± SEM.

To understand the progression of the FBR, the cell types present in the implanted tissue were monitored over time. Figure 3A shows a magnified section (×20) of the perimeter of the scaffold showing histological changes consistent with an acute immune response at 4 days post-implantation. The initial response of the material included the recruitment of many neutrophils, observable on the periphery of the scaffold. Cell density is reported as the total number of cells per field at 20× analyzed with ImageJ software (Figure 3B). At 7 days, there was a significant increase (*p* < 0.05) in the number of infiltrated cells in the AuNp + Alg scaffold (3136 ± 2003) compared to the without Np scaffold (1593 ± 772.8). At this time of implantation, increased metabolic activity was observed within the scaffold, characterized by regions with a population of dead cells, apparent cellular debris, and areas of myxoid degeneration, both close to the remnants of the biomaterial. Significant numbers of neutrophils remain on the periphery of the scaffold. The Alg–Cs scaffold shows a larger area referring to areas of cell necrosis; thus, a significant decrease in the cellular area occupied by the leukocyte infiltrate compared to the functionalized groups (Without Np: 12.54% ± 8.306%, AuNp: 25.82% ± 18.50%, AuNp + Alg: 42.25% ± 31.26%, *p* < 0.01) was observed (Figure 3C). At 25 days after implantation, macrophages represent the predominant cell type. FBGCs are observed located throughout the scaffold. There is a decrease in the number of FBGCs in the AuNp + Alg scaffold (Without Np: 9.8 ± 4.3, AuNp: 7.1 ± 2.6, AuNp + Alg: 5.8 ± 2.6, *p* < 0.05) (Figure 3D). Only a small number of neutrophils are observed around the remnant scaffold fibers. Finally, the epidermis tissue in contact with the scaffold contains the same structures as normal epidermal tissue.

**FIGURE 4:**
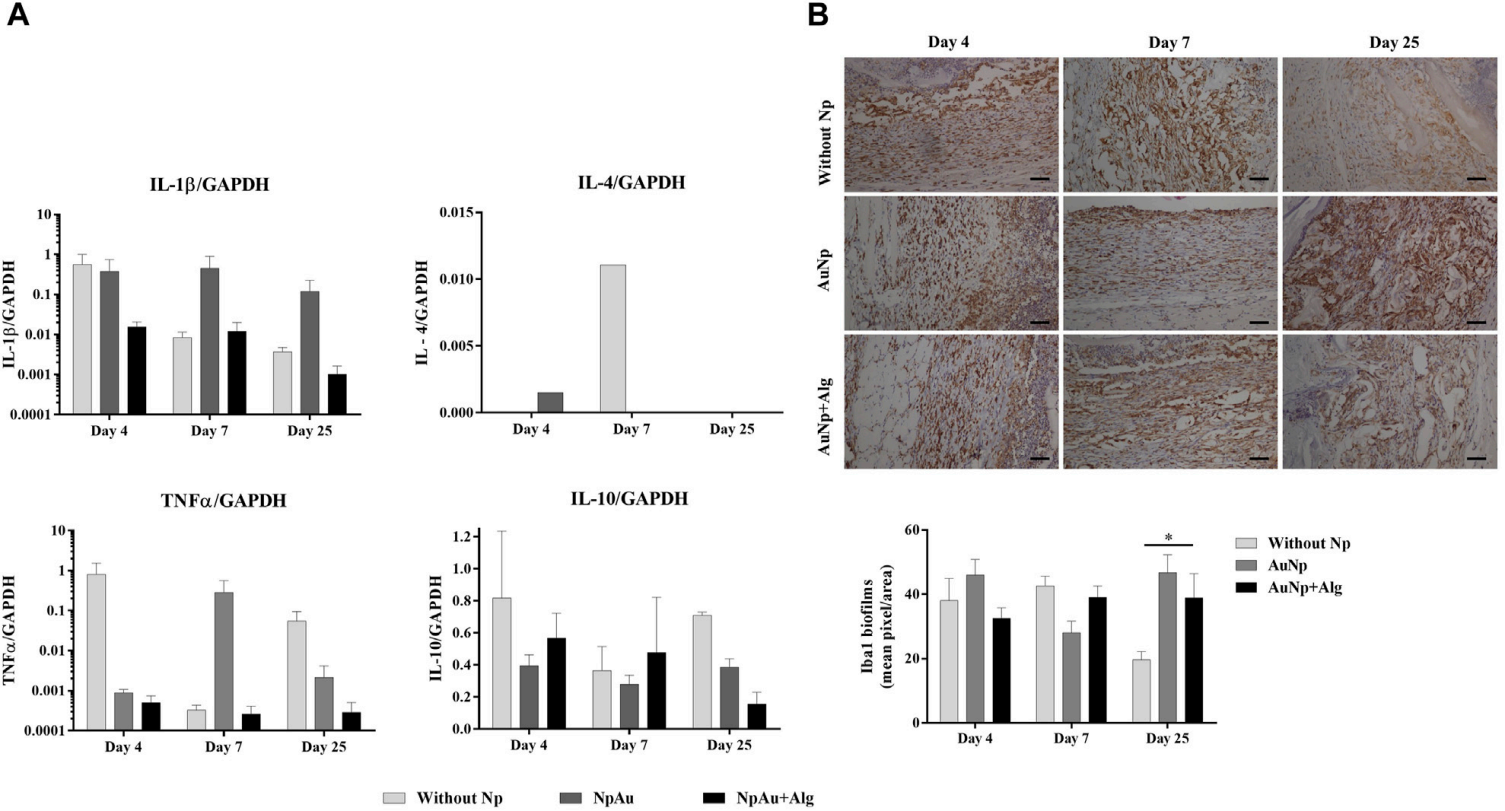
Inflammatory response. **(A)** qRT-PCR showing the levels of proinflammatory (IL-1β and TNFα) and anti-inflammatory (IL-4 and IL-10) cytokines. GAPDH was used as a housekeeping gene. **(B)** Representative immunohistochemical images and quantification of anti-Iba1 on the scaffolds at different points of time, (bar = 50 μm). Data are presented as mean ± SEM. (*) *p* < 0.05.

The real-time expression of genes for proinflammatory (IL-1β and TNFα) and anti-inflammatory (IL-4 and IL-10) cytokines was quantified (Figure 4A) from total RNA isolated from the implanted scaffolds. The expression of IL-1β tends to decrease over time in the groups without Np and AuNp + Alg. Despite not registering significant differences between groups, the expression of IL-1β in the AuNp + Alg group is apparently lower compared to that recorded in the experiment for 25 days. TNFα expression tends to increase in the without Np group at 4 and 25 and 7 days in the AuNp group. The low expression in the AuNp + Alg group remained unchanged throughout the study. In the case of anti-inflammatory cytokines, IL-4 expression was only recorded in the groups without Np and AuNp at 4 and 7 days. IL-10 expression tended to increase at 4 and 25 days in the without Np group and to decrease in the AuNp + Alg group at 25 days. No apparent changes were observed in the AuNp group.

In parallel, Iba1 expression was assessed with immunohistochemistry. Iba1 evidenced the level of inflammation in the entire scaffold and surrounding dermis tissue (Figure 4B). Iba1 expression was observed in all groups. The percentage of the positive area presented a tendency to increase in the presence of the scaffold in relation to the control group at 4 days postimplantation (control: 0.55% ± 0.33%, Without Np: 38.14% ± 23.76%, AuNp: 46.04% ± 24.22%, AuNp + Alg: 32.52% ± 16.48%). At 25 days, Iba1 expression was significantly higher in the NpAu scaffold compared to without Np (Without Np: 19.65% ± 8.96%, AuNp: 46.80% ± 25.85%, *p* < 0.05).

These results demonstrate that the FBR had resolved 25 days after implantation. The expression of anti-inflammatory cytokines and markers related to macrophage activation suggests a decrease in inflammation in Alg-coated and functionalized scaffolds.

### 3.3 Identification of fibrous capsule and collagen areas

**FIGURE 5:**
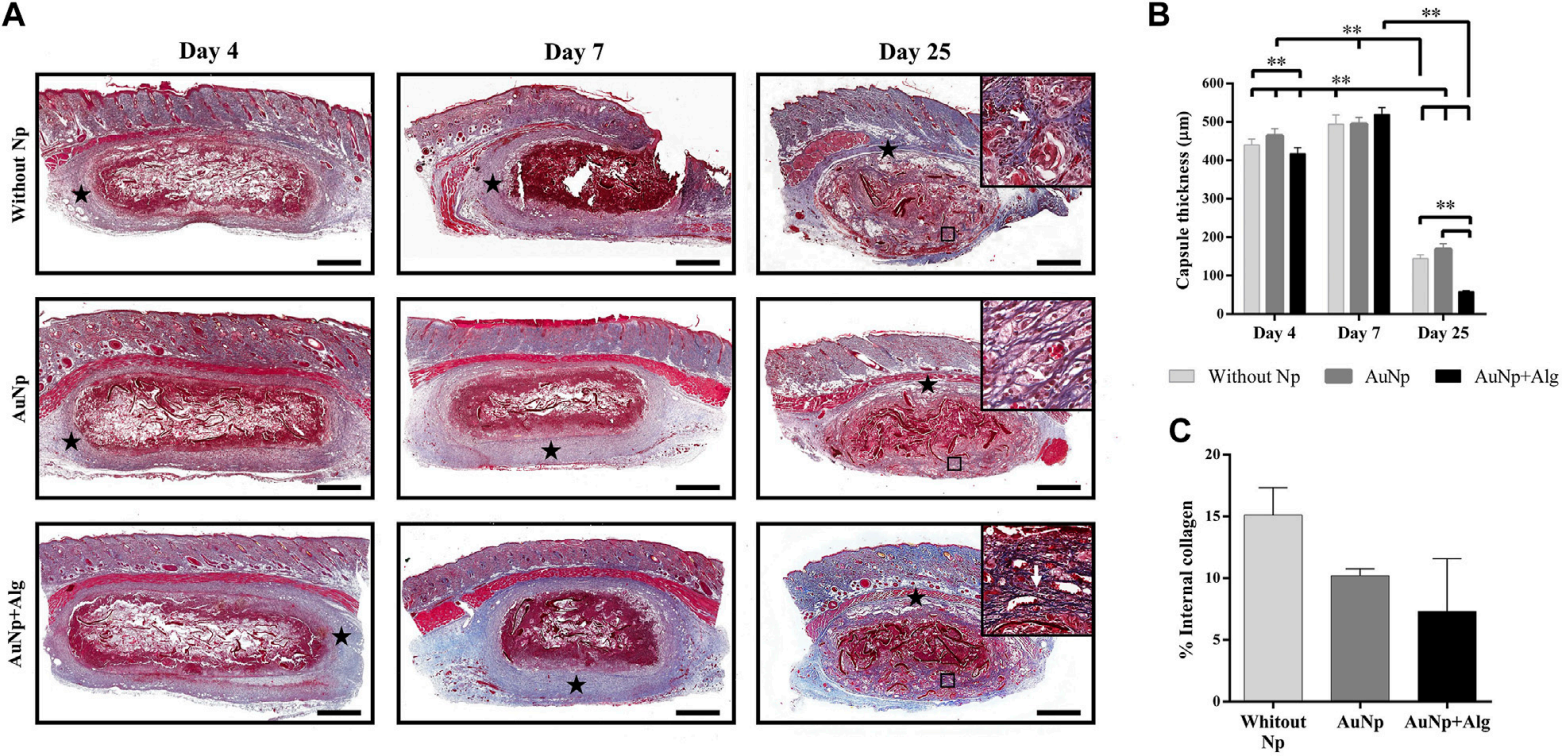
Representative photomicrographs of capsule thickness and internal collagen between groups over time **(A),** (Masson; bar = 1 mm). Framed regions were enlarged (Masson; x 200) to show collagen fibers. Identifiers: star: capsule; white arrow: collagen fibers. **(B)** Graphical representation of capsule thickness. **(C)** Graphical representation of collagen deposits inside the scaffolds. Data are presented as mean ± SEM. **p < 0.001.

MT-stained sections revealed the fibrous capsule formed around the implants. The response is similar among groups. The capsule thickness and internal collagen content are shown in Figure 5A. At 4 days, a capsule formed by lax connective tissue was observed, which subsequently became dense connective tissue at 7 days. The capsule thickness (Figure 5B) is similar among groups (440 ± 23 µm) at 4 days and tends to increase at 7 days (502 ± 14 µm). The capsule decreases at day 25 (Without Np: 150 ± 51 μm, AuNp: 169 ± 99 μm, AuNp + Alg: 58 ± 22 µm). Once the capsule has shrunk towards the ventral and dorsal region of the implant, granulation tissue is identified, characterized by a large number of blood vessels containing erythrocytes, which constitutes a vascularized interface.

At 25 days, collagen deposits were observed inside the scaffold, suggesting the presence of fibroblasts inside the material (framed regions in day 25, Figure 5A). The quantitative analysis did not show significant differences among groups, but there is a trend in the reduction of collagen content in the functionalized groups (Figure 5C) (Without Np: 15.11% ± 3.146%, AuNp: 10.19% ± 1.143%, AuNp + Alg: 7.309% ± 6.036%).

### 3.4 Macrophage polarization

**FIGURE 6:**
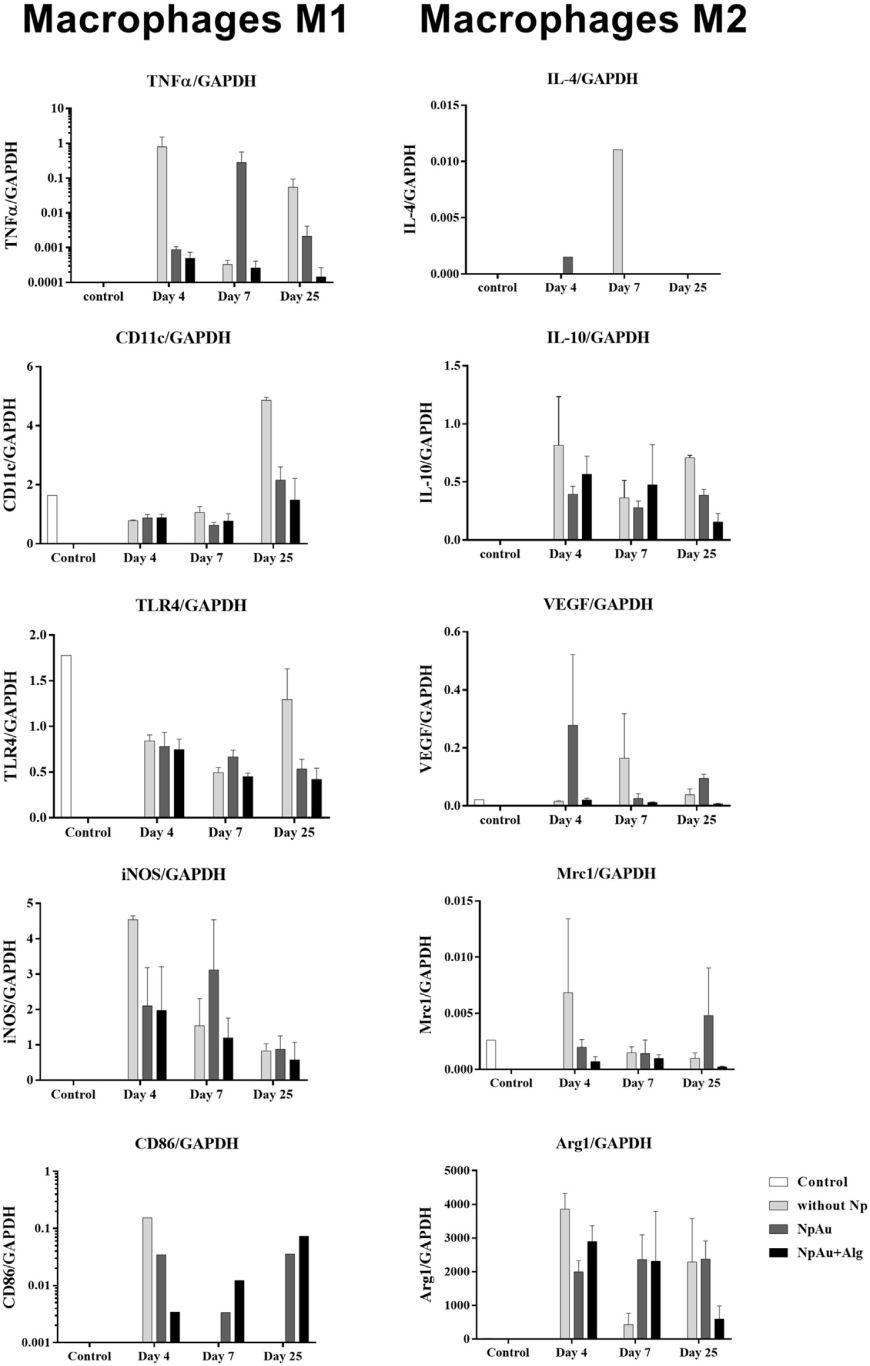
Macrophage profile. qRT-PCR values showing the expression of markers for the M1 (TNFα, CD11, TLR4, INOS, and CD86) and M2 phenotype (IL4, IL10, VEGF, Mrc1, and ARG1) in all scaffolds. Data are presented as mean ± SEM.

Real-time expression of M1 macrophage markers (TNFα, CD11, TLR4, INOS, and CD86) was quantified in the study groups (Figure 6). A trend towards a higher expression of TNFα was present in the NpAu group on day 7 and decreased on day 25. CD11 expression remained constant between groups at 4 and 7 days of the experiment, and a trend towards increased expression was observed at 25 days in the group without Np. TLR4 expression showed a trend to decrease with respect to the reference tissue (healthy skin) and remains constant over time. The expression of iNOS had a tendency to decrease over time and tended to be higher in the groups without the Np scaffold than in the functionalized groups at 25 days. During the analysis of the samples, a tendency to a decreasing expression of CD86 was observed within subjects.

The real-time expression of M2 macrophage markers (IL-4, IL-10, VEGF, Mrc1, and Arg1) is shown in Figure 6. Arg1 tends to decrease at day 4 in the without Np group, remains constant in the NpAu group, and tends to decrease as a function of time in NpAu + Alg.

### 3.5 Blood vessel formation

**FIGURE 7:**
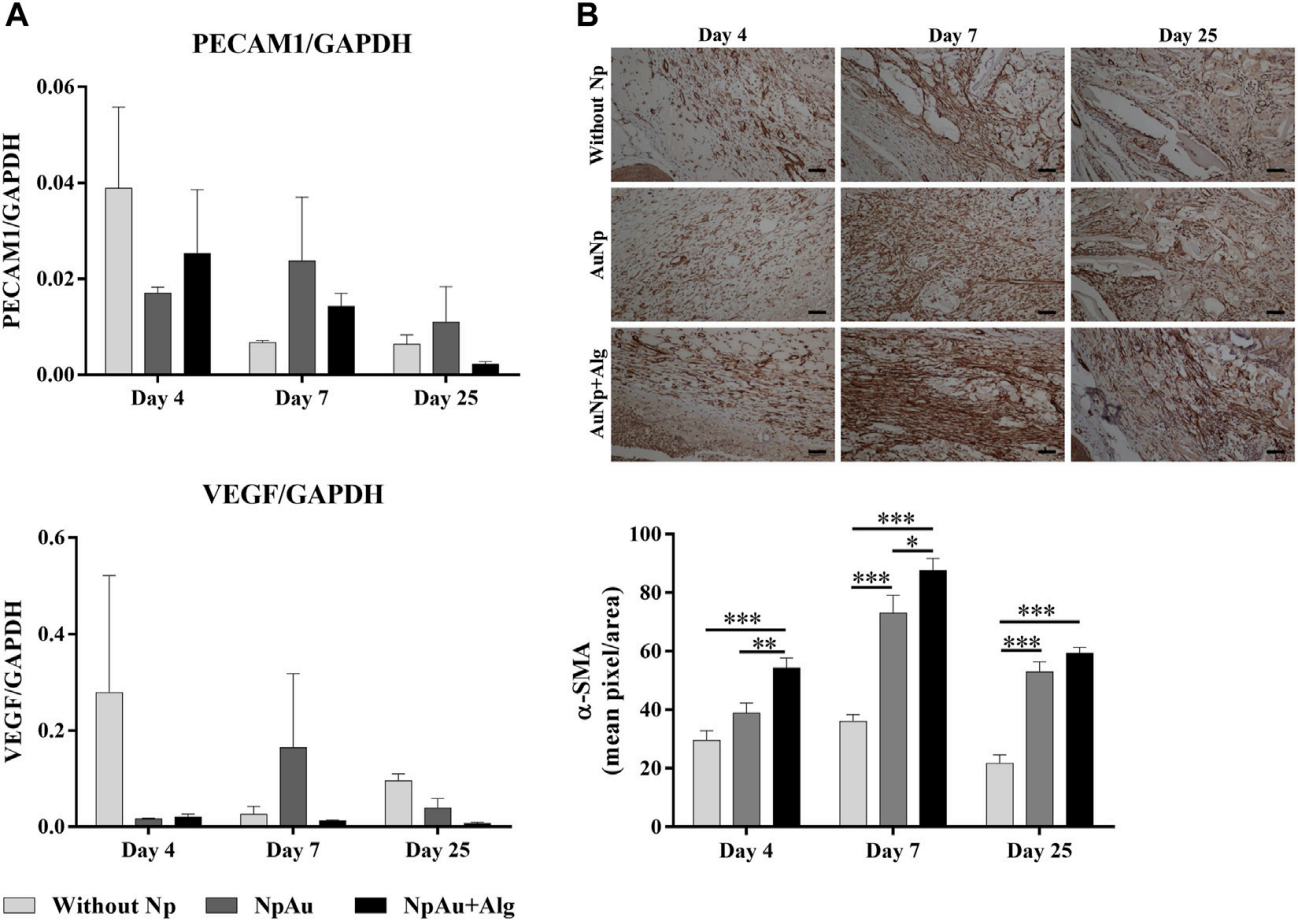
Angiogenic response. **(A)** qRT-PCR showing the levels of PECAM1 and VEGF, **(B)** Representative immunohistochemical images and quantification analysis of anti-αSMA on the scaffolds at different points of time, (bar = 50 μm). (*) *p* < 0.05, (**) *p* < 0.01, (***) *p* < 0.001. Data are presented as mean ± SEM.

As shown in Figure 7A, real-time expression was analyzed for PECAM-1 and VEGFa expression. No significant differences were observed among groups for eitther marker. PECAM-1 expression showed a tendency to increase at 4 days postimplantation and to decrease toward the end of the experiment. VEGFa expression tended to be higher in the without Np scaffold at 4 and 25 days. At 7 days, an increasing trend in VEGFa expression was identified in the NpAu group.

The expression of α-SMA (smooth muscle actin, vascular marker) using immunohistochemistry was observed in blood vessels in all three groups (Figure 7B). Quantitative analysis indicated that α-SMA expression was significantly higher in the functionalized groups compared to the without Np scaffold, at 4 days. A significant increase (*p* < 0.05) of α-SMA was identified at 7 and 25 days in the AuNp and AuNp + Alg groups with respect to the Without Np group (D7, Without Np: 36% ± 8.23%, AuNp: 73.03% ± 29.86%, AuNp + Alg: 87.57% ± 17%; D25 Without Np: 21.71% ± 9.53%, AuNp: 52.97% ± 16.76%, AuNp + Alg: 59.28% ± 7.01%).

## 4 Discussion

The novelty of our scaffold is related to its composition (alginate 0.75% and chitosan 1.25% w/v) and its manufacturing process, which includes the synthesis and incorporation of metallic gold nanoparticles, without and with an alginate cover, which was previously reported by our group (Beltran-Vargas et al., 2022). Additionally, it is important to evaluate the effect of adding an alginate coating to gold nanoparticles on an alginate -chitosan scaffold, to generate a highly vascularized platform. In the area of ​​tissue engineering, it has been documented that the efficiency of therapy increases in strategies that have been functionalized with organic or inorganic molecules. However, the number of functionalized proposals is low in relation to non-functionalized proposals. Therefore, our scaffold meets the current needs in tissue engineering. Limited studies have focused on how biomaterials affect host response, such as inflammation and immune modulation. Given that the functionalization of biomaterials results in an improved representation of the microenvironment for cell culture, the study of biocompatibility in *in vivo* models is of interest to research groups related to the use of non-cytotoxic conductive natural biomaterials.

The success of using biomaterials in TE depends on their ability to not generating an adverse effect on the host organism, such as cytotoxicity, mutagenicity, carcinogenicity, and immunogenicity (Raut et al., 2020). According to macroscopic observations (Figure 1) and histological analysis, our scaffolds showed adequate biocompatibility through integration with host tissue, cell recruitment and release of anti-inflammatory cytokines, blood vessel enlargement, and granulation tissue development within 25 days.

The use of Alg and Cs scaffolds, individually and in combination, has been reported in TE (Farshidfar et al., 2023; Kim et al., 2023). The combination of these biomaterials results in the formation of a complex that can swell in the presence of body fluids (e.g., exudates) (Hao et al., 2021), in addition to modulating the inflammatory phase (Zhu et al., 2020; Soriente et al., 2022), stimulating fibroblast proliferation and accelerating wound healing (Caetano et al., 2015), as well as improving scar tissue quality (Breder et al., 2020). However, the null electrical properties of these scaffolds represent a limitation to replicating the characteristics of various conductive tissues. Conductive scaffolds are often used in TE to create an electrical interface with cells and enable tissue stimulation. This is important during the development of electrically active tissues such as cardiac muscle and nerve tissue. Natural and synthetic scaffolds with Au incorporation exhibit improved cell viability, binding, and proliferation (Shevach et al., 2014; Baranes et al., 2016; Ghaziof et al., 2022). When evaluating *in vivo* models, Au incorporation in scaffolds promotes proper communication of the graft with the host tissue (Dong et al., 2020).

The incorporation of coated nanoparticles into TEs has been recently explored. Coating Nps with natural materials has been reported to result in improved stability (Sood et al., 2017) and interaction with biological systems *in vitro* (Shen et al., 2019). However, little evidence points to mechanisms associated with their application in animal models. In this work, we evaluated the biocompatibility of alginate-coated AuNp-functionalized scaffolds (AuNp + Alg). Our scaffolds generated the typical FBR (Figure 2), reported by (Bushkalova et al., 2019) and by (Ribeiro et al., 2021); with an increase in cellular infiltrates, reduction in capsule size, and the time to resolution of the inflammatory reaction. The scaffolds used in this work are highly porous (Beltran-Vargas et al., 2022), and it has been reported that scaffolds with these characteristics show less fibrous encapsulation and greater integration of the implant compared to biomaterials with less porosity; in addition to promoting high levels of cellular infiltration and angiogenesis (Whitaker et al., 2021).

Cell infiltration allows for examining the ability of cells to migrate and grow within the scaffold over time. It has been reported that high values of porosity and pore size between 30 and 40 µm can induce increased cell adhesion and promote, in macrophages, an M2 phenotype (Whitaker et al., 2021; Hernandez and Woodrow, 2022). *In vitro*, our scaffolds present high permeability, porosity, and swelling (Beltran-Vargas et al., 2022), which promotes cell recruitment of up to twice the number of cells recorded in non-functionalized scaffolds at 7 days postimplantation (Figure 3B). Similar results were observed in a previous investigation (Dulany et al., 2020), where functionalization with cerium oxide nanoparticles in a synthetic scaffold increased cellular infiltration by 33% with respect to its reference group. In addition, increased cellular infiltrates and decreased duration of acute inflammatory response are associated with early resolution of FBR (Barone et al., 2022). Our results show a decrease of about half the number of infiltrating cells at 25 days compared to their initial values (Figure 3B). Resolution of the inflammatory response in our scaffolds occurs at about 4 weeks. This is a clear improvement over nonfunctionalized chitosan scaffolds, which show a longer resolution of up to 8 weeks (Modulevsky et al., 2016). The distribution of nuclei along the biomaterial, in relation to the tissue events present within the scaffold, may indicate the degree of inflammatory response. In our study, incorporating AuNp + Alg increases the percentage of occupied area within the scaffold, thus reducing the areas of necrosis (Figures 3A, C). Similar results were reported in previous studies (Snider et al., 2022; Cheng et al., 2023). *In vitro*, reduction of cell death by apoptosis in chondrocytes was observed when a decellularized matrix functionalized with 20 nm AuNp was used (Snider et al., 2022). Moreover, the presence of FBGC on and around implanted biomaterials is considered evidence of a chronic inflammatory response of the host tissue to these materials (McNally and Anderson, 2015). In our study, the NpAu + Alg group presented a decrease in FBGC density (Figure 3D), indicating increased biocompatibility in functionalized scaffolds.

Cytokine induction can be used to assess the intensity of immune reactions of biomaterials since biocompatibility is reduced when biomaterials induce very high amounts of cytokine expression (Ding et al., 2007). Thus, the low detection of TNFα and IL-4, shown in Figure 4, suggests that subdermal implantation of Alg/Cs scaffolds without/with NpAu elicits a mild immune reaction. Previous studies have reported that the use of NpAu in combination with other materials decreases the infiltration of inflammatory cells and the level of proinflammatory mediators such as iNOS, COX-2 and cytokines such as TNF-α, IL-1β, IFN-γ and IL- 6; In addition, it increases the expression of anti-inflammatory cytokines such as TGF-β, IL-10 and IL-4 (Park et al., 2019; Mahmoudi et al., 2022).

The design and functionalization of the scaffold impact the expression of the immune response produced after implantation, resulting in variations in the size of the capsule surrounding the biomaterial. The capsule size recorded in our investigation, as shown in Figure 5B, is smaller than that reported by other studies (Divakar et al., 2020), where a capsule thickness of 3 mm was observed around scaffolds made from unfunctionalized collagen. Results similar to those obtained in our investigation were also reported (Dulany et al., 2020; Camarero-Espinosa et al., 2022). The significant reduction in capsule size is usually associated with an increase in pore size (Barone et al., 2022) and modifications in the functional groups of the biomaterial (Jeong et al., 2017). Pore morphology, including size, shape, and microstructure also affect the balance between fibrous encapsulation and tissue integration (Whitaker et al., 2021).

Together with granulation tissue, fibrosis represents a regeneration phase during the reduction of inflammation, with the deposition of extracellular matrix and collagen fibers by fibroblasts within the scaffold (Hernandez and Woodrow, 2022). Our investigation showed increased collagen deposition within the biomaterial in unfunctionalized scaffolds (without Np) (Figure 5C). However, adding Np did not interfere with the resolution of FBR in our scaffolds.

The activity of macrophages and fibroblasts is closely related (Witherel et al., 2019). Macrophages are responsible for releasing proinflammatory cytokines related to NF-κB activation and matrix metalloproteinase production, whereas fibroblasts are responsible for stimulating FGF synthesis. Cs has been described to be analogous to glycosaminoglycans, which stimulate the FGF-2 signaling pathway, chemically bind to it, and facilitate interaction with its cellular receptors on endothelial cells of various tissues (Muzzarelli, 2009). Recently, it has been shown that Cs can stimulate the production of anti-inflammatory cytokines and growth factors in macrophages, which induce fibroblast activity, thus favoring the resolution of inflammation and tissue repair (Muxika et al., 2017; Ribeiro et al., 2021).

During FBR, macrophages initially assume an M1 phenotype that promotes inflammation by releasing inflammatory cytokines (IL-6, IL-12, and TNFα), reactive oxygen species, and antimicrobial peptides. After the acute inflammatory phase subsides, the macrophage population shifts to an M2 phenotype. M2 macrophages are characterized by the secretion of anti-inflammatory mediators (IL-10) and growth factors (PDGF and TGF-β) that aid in tissue healing by stabilizing angiogenesis (Karkanitsa et al., 2021; Martin and García, 2021). In our study, we observed the coexpression of M1 and M2 markers (Figure 6), which has usually been reported in FBR induced by scaffolds (Witherel et al., 2019).

The M1 phenotype is generally identified by the expression of surface markers and co-stimulatory molecules such as CD86 and intracellular molecules such as iNOS (Martin and García, 2021; Wei et al., 2021). In our work, markers such as TNFα and CD86 used to characterize M1 macrophages are expressed in small amounts (see scale of TNFα and CD68 in Figure 6). iNOS had a tendency to decrease throughout the experiment in all study groups, suggesting a resolution of the immune response (Figure 6). It has been documented that chitosan inhibits TNFα production and induces IL-8 expression, which promotes angiogenesis and neutrophil migration (Mori et al., 1997).

The M2 phenotype is characterized by the expression of surface markers and intracellular arginase 1. In our study, markers such as IL-10, Mrc1, and Arg1 are present on the without Np scaffolds and the functionalized scaffolds, thus indicating the presence of the M2 phenotype (Figure 6). Natural biomaterials promote the positive regulation of IL-4, which is released to limit the degree of injury (Karkanitsa et al., 2021). However, in our study, IL-4 expression was not observed in all groups over time. Similar results have been also reported (Vasconcelos et al., 2015; Soriente et al., 2022).

In addition to capsule formation, cell infiltration, and FBGC formation, the protrusion of neovascular sprouts into the biomaterial and surrounding tissue is a significant feature of FBR. Fibroblasts form a fibrous capsule around the biomaterial, which insulates it from the rest of the body and produces extracellular matrix components. In the early stages of FBR, the cells of the cellular infiltrate are likely to encounter locally compromised oxygen pressure (Capuani et al., 2022). Hypoxia activates macrophages to induce hypoxia-inducible transcriptionally active factors, which induce the expression of angiogenic factors such as VEGF, PDGF, adrenomedullin, angiopoietin 2, and others (Boomker et al., 2005). On the other hand, at later stages in FBR, the formation of new blood vessels facilitates the arrival of more inflammatory cells, which may aggravate the inflammatory response. Thus, early angiogenesis is beneficial to ameliorate the response and allow for cell migration within the scaffold (Parlani et al., 2022). Our results show a trend in increased VEGF and PECAM1 expression at the onset of the inflammatory response in all three groups (Figure 7A), while vessel detection by αSMA increases at week 7 and is higher in the functionalized groups (Figure 7B). Porous scaffolds have been documented to promote blood vessel formation. It has been described that the number and diameter of blood vessels are enhanced in the presence of scaffolds made with pores larger than 150 µm (Walthers et al., 2014; Eichholz et al., 2022). On the other hand, in bone tissue engineering, *in vivo* studies showed that the use of various biomaterials functionalized with AuNPs induced angiogenesis at the defect site (Samadian et al., 2021).

In sum, the absence of exacerbated reactions in the host tissue allows us to confirm that the implanted scaffolds are biocompatible. Therefore, AuNp-functionalized scaffolds offer several benefits for TE use while maintaining communication with the host tissue. Should be interesting to test our scaffolds in other organs and tissues. An evaluate other pro- and anti-inflammatory markers with a larger sample.

## 5 Conclusion

In this study, we evaluated *in vivo* the biocompatibility of scaffolds made from alginate and chitosan (Alg/Cs), functionalized with AuNp and AuNp + Alg. The results suggest that the combination of Alg/Cs with gold nanoparticles forms a scaffold that can swell in the presence of body fluids, modulate the inflammatory process, stimulate fibroblast proliferation and collagen fiber production, promote blood vessel development, and improve scar tissue quality. These scaffolds have the potential to be used for the incorporation of a cellular component for use as regenerative therapy.

## Data Availability

The original contributions presented in the study are included in the article/supplementary material, further inquiries can be directed to the corresponding author.
